# Clinicopathologic Features and Molecular Biomarkers as Predictors of Epidermal Growth Factor Receptor Gene Mutation in Non-Small Cell Lung Cancer Patients

**DOI:** 10.3390/curroncol29010007

**Published:** 2021-12-24

**Authors:** Lanlan Liu, Xianzhi Xiong

**Affiliations:** Department of Respiratory and Critical Care Medicine, NHC Key Laboratory of Pulmonary Diseases, Union Hospital, Tongji Medical College, Huazhong University of Science and Technology, 1277 Jiefang Rd, Wuhan 430022, China; m201975688@hust.edu.cn

**Keywords:** epidermal growth factor receptor, non-small cell lung cancer, predictor

## Abstract

Lung cancer ranks first in the incidence and mortality of cancer in the world, of which more than 80% are non-small cell lung cancer (NSCLC). The majority of NSCLC patients are in stage IIIB~IV when they are admitted to hospital and have no opportunity for surgery. Compared with traditional chemotherapy, specific targeted therapy has a higher selectivity and fewer adverse reactions, providing a new treatment direction for advanced NSCLC patients. Tyrosine kinase inhibitors of epidermal growth factor receptor (EGFR-TKIs) are the widely used targeted therapy for NSCLC patients. Their efficacy and prognosis are closely related to the mutation status of the EGFR gene. Clinically, detecting EGFR gene mutation is often limited by difficulty obtaining tissue specimens, limited detecting technology, and economic conditions, so it is of great clinical significance to find indicators to predict EGFR gene mutation status. Clinicopathological characteristics, tumor markers, liquid biopsy, and other predictors are less invasive, economical, and easier to obtain. They can be monitored in real-time, which is supposed to predict EGFR mutation status and provide guidance for the accurate, individualized diagnosis and therapy of NSCLC patients. This article reviewed the correlation between the clinical indicators and EGFR gene mutation status in NSCLC patients.

## 1. Introduction

Lung cancer ranks as the most common cancer and a significant cause of cancer death globally [1]. Non-small cell lung cancer (NSCLC) is the predominant type of lung cancer, accounting for about 80–85%. NSCLC mainly consists of lung adenocarcinoma (ADC), lung squamous cell carcinoma (SCC), adenosquamous carcinoma (ASC), etc. [2,3,4]. More and more driver genes have been found in recent years, and the epidermal growth factor receptor (EGFR) gene is the most common one in NSCLC patients. EGFR gene mutation frequency varies in different countries and regions around the world, with the highest frequency (49.1%) in the NSCLC patients of Asian countries compared with other continents (11.9–33.0%) [5]. EGFR tyrosine kinase inhibitors (EGFR-TKIs) are widely applied to the advanced NSCLC, which lays a foundation for promoting individualized and targeted therapy. Its therapeutic effect takes a close relationship with the EGFR gene mutation status. Currently, the standard methods for detecting EGFR gene mutations are direct sequencing, amplification refractory mutation system (ARMS), etc., which have high requirements for tissue specimens. The majority of NSCLC patients at first diagnosis are in stage IIIB~IV and have no opportunity for surgery, so it is challenging to obtain tumor tissue specimens. The sample size of tissue obtained by fine needle aspiration biopsy is small, which may decrease the detection rate of EGFR gene mutation. Other specimens such as blood and pleural effusion (PE) are sometimes unsatisfactory, limiting the clinical application of genetic testing. The indicators, including clinicopathological features, serum tumor markers, liquid biopsy, and others which are less invasive, economical, easier to obtain, and monitored in real-time, have been proven to be associated with EGFR gene mutation. As such, they are supposed to reflect the status of EGFR gene mutation. This article will review the relevant research in recent years.

## 2. Demographics

### 2.1. Gender and Smoking History

There is evidence of the correlation between EGFR mutation status and patients’ demographics (mainly including gender, smoking history, and age), but results are conflicting. In terms of gender and smoking history, a large number of studies agreed that in NSCLC, EGFR mutation frequency in women and non-smoking patients was higher than in men and smokers [6,7,8]. Demographic characteristics and EGFR mutation rates are shown in Table 1. Females and non-smokers were essential predictors of EGFR mutation [9]. However, Ess et al. [10] reported that in non-smoking patients, the EGFR mutation frequency between different genders had no significant difference (*p* = 0.2). Smoking index (number of cigarettes per day × number of years of smoking) was able to predict the status of EGFR mutation with a negative correlation, indicating that the smaller the smoking index was, the greater the probability of EGFR mutation was [2]. According to different mutation sites, the EGFR gene mutation can be classified into various mutation subtypes. Exon 19 deletion (19-Del) and exon 21 L858R mutation (L858R) are considered the most common mutation types, accounting for 49.7% and 50.3%, respectively [11]. Wei et al. [12] observed no significant differences in gender and smoking status between the two common mutations 19-Del and L858R. In a recent meta-analysis [5] for NSCLC patients worldwide, the percentage of male patients was a significant covariate for the overall EGFR mutation but not for the mutation subtypes. With the increase of the percentage of males, the EGFR mutation frequency decreased.

### 2.2. Age

There is some controversy about the relationship between EGFR mutations and age. Many investigations reported age was not related to EGFR mutation. When NSCLC patients were divided into younger and older groups (different studies chose different cutoff points for age, namely 60, 62, 65, etc.), no significant difference existed in the rates of EGFR mutation among different groups [4,7,8]. The median ages were also comparable between the EGFR-mutated and wild groups [6,9,23]. In a recent meta-analysis, age was not considered as a covariate for the overall EGFR mutation [5]. Some studies take a different view on the relationship between EGFR mutations and age. Vallee et al. [24] reported that EGFR-mutated patients were older than EGFR-wild patients (median age, 71 vs. 63 years, *p* < 0.001). Some studies found that the mutation rate was higher in patients under 60 years of age than patients over 60 years, and the difference in mutation rates was statistically significant [25,26]. Recently, Lemine et al. [20] found that NSCLC patients with EGFR mutations were younger than patients with wide-type EGFR in Morocco (mean age, 61 vs. 62 years, *p* = 0.041). A Caucasian population study found no difference in EGFR mutation frequency among age groups in males. Nevertheless, in females, the frequency was higher in the older patients than in the younger group (cutoff age, 65 years) (22.6% vs. 12.2%, *p* < 0.001) [3]. Zhang et al. [11] found that the 19-Del was more common than the L858R mutation in patients younger than 50 (*p* < 0.001). Further, in a subgroup analysis of age, the 19-Del mutation rate was higher than the L858R mutation in patients aged between 21 to 30, 31 to 40, 41 to 50, and 51 to 60, while the 19-Del rate was lower than the L858R mutation in patients aged 61 to 70, 71 to 80, and 81 to 90. It confirmed the different distribution of EGFR mutant subtypes in various age groups.

## 3. Pathological Features

### 3.1. Pathological Types

The most common pathological type of NSCIC is ADC, followed by SCC, ASC, etc. These three types account for more than 95% of all NSCLC, and ADC is much more than non-adenocarcinoma [7,10]. EGFR mutation rate varies in different pathological types, and the mutation in ADC was more than that in non-adenocarcinoma [6,27]. Wang et al. [9] revealed that the difference in mutation frequency among pathological types such as ADC, SCC, and ASC was statistically significant (53.87%, 16.67%, and 50.00%, *p* < 0.001). The pathological type was an independent predictor for the EGFR mutation status. That is, patients with ADC are more likely to harbor EGFR mutation [9]. Moreover, the subgroup analysis of pathological types found no statistically significant difference between exon 19 and 21 mutations in ADC, SCC, and ASC [11,12].

Furthermore, multiple studies have discussed the relationship between EGFR mutation and ADC histological subtypes, but the results are inconsistent. ADC is divided into preinvasive lesions, minimally invasive adenocarcinoma (MIA), invasive adenocarcinoma (IAC), and variants of invasive adenocarcinoma, among which IAC is the most common subtype. Preinvasive lesions consist of atypical adenomatous hyperplasia and adenocarcinoma in situ (AIS). EGFR mutations are related to histological subtypes of ADC, and the mutation rate in IAC is the highest [6,17,25]. In different subtypes of IAC, acinar predominant adenocarcinoma (APA) has the highest EGFR mutation frequency, up to 47.4~81.7% [16,23,28,29]. Girard et al. [14] revealed that in both East Asia and non-Asia, EGFR mutations were common in papillary predominant adenocarcinoma (PPA) (30%) but rare in APA or solid predominant adenocarcinoma with mucin production (SPA). A recent study [30] also pointed out that PPA was associated with a high rate of EGFR mutations (23.7% vs. 76.3, *p* = 0.038). Shim et al. [13] analyzed 107 ADC patients and found the mutation in micropapillary predominant adenocarcinoma (MPA) was more common than that in other histological subtypes (83.3% vs. 46.3%, *p* = 0.02). In comparison, the mutation was less common in SPA than in other subtypes (28.6% vs. 55.8%, *p* = 0.03). Sun et al. [31] also confirmed the above view and found no significant difference in mutation frequencies among other histological subtypes. A systematic review of 27 studies [32] reported that the EGFR mutation rate was the highest in lepidic predominant adenocarcinoma (LPA) (50.7%), while SPA (*p* < 0.01) and invasive mucinous adenocarcinoma (IMA) (*p* < 0.01) were associated with the EGFR-wild status. Further, in gender analysis, the EGFR mutation was associated with AIS, MIA, and LPA subtypes in male patients, but in women, it was related with APA and PPA subtypes [33]. Another study found similar rates of EGFR mutation between MIA and IAC (*p* = 0.334) [34].

### 3.2. Lymph Node Metastasis

Some studies concluded that EGFR mutation had no significant correlation with lymph node metastasis in NSCLC patients [19,25]. Xu et al. [15] found that distant lymph node involvement was associated with EGFR mutation, but local nodal involvement was not the significant predictive factor for EGFR mutation in ADC. Dong et al. [16] also reported that lymph node metastasis was not the related predictor of EGFR mutation in adenocarcinoma (*p* = 0.257). On the contrary, some studies held different views about it. In a single-center study involving 1506 NSCLC patients with different TNM-N stages, the rates of EGFR-TKIs sensitive mutations were 37.5%, 45.7%, 39.3%, and 32.7%, respectively, in the stages of N0, N1, N2, and N3 (*p* = 0.036). However, the mutant rates in the N0–N3 stages showed no noticeable change trend [12]. The study of non-squamous NSCLC patients who received surgical resection, which evaluated clinicopathologic features of EGFR mutation, suggested that lymphatic permeation was lower in tumors harboring EGFR mutations (37.0% vs. 41.9%, *p* < 0.001) [22]. In a study involving 827 ADC patients, EGFR mutation was related to a low frequency of lymph node metastasis (*p* = 0.006) [35]. As for the different EGFR mutation subtypes, patients with 19-Del (13.6%) suffered from a higher risk of lymph node metastasis than with wild-type (9.1%) and L858R (5.6%), although with no statistical difference (*p* = 0.119) [36]. Zhang et al. [11] reported that the incidences of 19-Del and L858R varied in different N stages (*p* < 0.001), and the N stage can predict the subtype of EGFR gene mutations, which may help select the individual therapy for EGFR-mutated patients. The mechanism deserves further study.

### 3.3. Degree of Differentiation

Studies have shown mixed results of the correlation between the degree of differentiation and EGFR mutation. Gu et al. [18] pointed out that EGFR mutation rates in poor, moderate, well, and undefined differentiation were 17.8%, 35.8%, 50.0%, and 37.7%, respectively, showing no relevance (*p* = 0.081). Some studies suggested EGFR gene mutation was associated with tumor differentiation. Levy et al. [37] reported a study on the histologic grade and EGFR mutation in 277 patients with NSCLC. They demonstrated that for the grade I (well differentiated) and II (moderately differentiated) patients, the frequency of EGFR mutation was 38.6% and 36.6%, respectively, but 6.7% for the grade II-III (moderately to poorly differentiated), and 11.3% for grade III histology (poorly differentiated) patients (*p* < 0.0001). They concluded that histologic grade is a valuable predictor for EGFR mutation. The well differentiated and moderately differentiated patients were three times more likely to harbor mutations than moderately to poorly differentiated patients and poorly differentiated patients. A total of 358 NSCLC patients without smoking history were divided into three groups according to the differentiation degree, which comprised poorly, moderately, or well differentiated groups. By comparing the differentiation degree of patients harboring EGFR mutation or not, well differentiated tumors in patients with EGFR mutation are more common than those in wild-type patients (*p* < 0.05) [38]. As for ADC, EGFR mutation was also frequently identified in well–moderately differentiated tumors [15,39]. As for different mutation types, there was no statistical difference in differentiation degree between 19-Del and L858R mutations [11].

### 3.4. Tumor Mutation Burden

Tumor mutation burden (TMB) is the total number of mutations in the exon coding region of tumor genes per megabase (Mb), including replacement, insertion, or deletion mutations [40]. TMB has an effect on the occurrence and progression of tumors. It is a new genetic marker that can reflect the overall status of tumor gene mutations. TMB has been widely used in screening the benefit population from immune checkpoint inhibitor therapy and efficacy prediction for NSCLC patients [41]. It has also been studied in EGFR-mutated NSCLC patients [42]. When patients with ADC were segmented into low-level (TMB < 10) and high-level (TMB ≥ 10) groups according to TMB, patients without EGFR mutation tended to have a higher level of TMB (OR = 4.707) [43]. Further, in the analysis of different EGFR mutation types, they found patients with 19-Del, L858R, and uncommon mutations had comparable TMB levels (*p* = 0.611). However, other studies revealed that the TMB level was lower in the EGFR 19-Del mutation than in the L858R mutation [44,45]. In targeted therapies, high TMB levels may be related to increased drug resistance pathways or clinical drug resistance due to tumor subcloning. Offin et al. [45] divided NSCLC patients, who harbored EGFR mutations and were treated with EGFR-TKIs, into low (TMB ≤ 2.83 mutations/Mb), intermediate (2.84 < TMB < 4.85 mutations/Mb), and high (TMB > 4.85 mutations/Mb) groups. The result indicated that TMB level was negatively correlated with the clinical benefit of EGFR-TKIs treatment for EGFR-mutant NSCLC patients. TMB is easy to calculate, repeat, and closely related to immunotherapy, targeted therapy effect, and prognosis. More exploration of TMB is needed in the field of EGFR-mutant NSCLC.

### 3.5. Immunohistochemistry

#### 3.5.1. Thyroid Transcription Factor-1

Thyroid transcription factor-1 (TTF-1), a member of the NKX2 transcription factor family, is mainly distributed in thyroid follicular cells, respiratory tract type II alveolar epithelium, etc. It has high selectivity for tumors of thyroid and lung origin. After excluding thyroid cancer, the expression level of TTF-1 can be used as a specific marker for the ADC and a routine immunohistochemical indicator of lung cancer [46]. In recent years, many observational studies have revealed a strong positive correlation between EGFR gene mutation status and TTF-1 [3,12,24,47,48]. In 1089 Chinese NSCLC patients, 52.41% of TTF-1-positive patients were accompanied by EGFR mutation, while only 14.66% of TTF-1-negative patients were with mutation (*p* < 0.001) [9]. A study of 909 North Indian NSCLC patients found a similar trend between EGFR mutations and TTF-1 [21]. A total of 90% of EGFR mutant NSCLC patients were with TTF-1 positive expression, indicating that TTF-1 had high sensitivity and negative predictive value (NPV) for EGFR mutation (90% and 87%, respectively). Vallee et al. [24] also pointed out that TTF-1 was positive in almost all EGFR-mutated patients (98.0%), and the sensitivity and specificity of TTF-1 in detecting EGFR mutation in lung adenocarcinoma were 99.1% and 36.4%, respectively [47]. A meta-analysis involving 9764 NSCLC patients [49] showed that the incidence of EGFR mutation was significantly higher in patients with TTF-1 overexpression than that in TTF-1 negative patients (OR = 5.19). Further, in the subgroup analysis, TTF-1 expression was significantly related to EGFR gene mutation in East Asian (*p* < 0.00001), European (*p* < 0.01), exon 19 (*p* < 0.00001), exon 21 (*p* = 0.04), male (*p* = 0.0009), and female (*p* < 0.0001) patients. Only 9.8% of NSCLC patients without TTF-1 expression harbored EGFR mutations, and the lack of TTF-1 expression had a high NPV (90.2%) for EGFR gene mutation. Kim et al. [49] revealed higher OR in females compared to males (OR, 4.87 and 3.34, respectively), while Nakra et al. [21] concluded comparable results between men and women (OR, 3.91 and 3.19, respectively). Wei et al. [12] observed a similar trend in TTF-1 expression between 19-Del and L858R (*p* = 0.367). To sum up, when EGFR mutation status is difficult to detect in time, TTF-1 may be an alternative predictor.

#### 3.5.2. Napsin A

Napsin A is a new gastric enzyme-like aspartic acid protease, which is significantly expressed in the lungs and kidneys. Napsin A had a very high sensitivity (91.9%), PPV (90.3%), and diagnostic accuracy (91.0%) for lung adenocarcinoma [50]. As an immunohistochemical indicator in diagnosing lung adenocarcinomas, Napsin A can distinguish the primary lung adenocarcinoma from metastatic lung tumors. The EGFR gene mutation frequency in patients with positive Napsin A expression was 52.42%, higher than that in patients with negative Napsin A expression (18.97%) (*p* < 0.01). Moreover, a similar trend exists in the study of lung adenocarcinoma [9]. At present, there are few studies on the correlation between Napsin A and EGFR mutation. Therefore, more clinical studies are expected to explore the relationship between the two.

## 4. Serum Tumor Markers

### 4.1. Carcinoembryonic Antigen

Clinically, carcinoembryonic antigen (CEA) is a routine serum tumor marker (STM). Abnormally elevated CEA level is common in adenocarcinoma and advanced cancer. Multiple pieces of research have shown that EGFR mutation status in NSCLC is related to CEA level [4,17,51,52]. Serum tumor markers and EGFR mutation rates are shown in Table 2. Some studies suggested that patients with a high CEA level had a higher rate of EGFR mutation than patients with a low CEA level. Gao et al. [4] demonstrated that the median value of CEA level was lower in EGFR-wild patients than the mutant patients (6.0 vs. 12.5 ng/mL, *p* = 0.001). EGFR mutation frequency increased in patients with a high level of CEA compared to patients with a low level of CEA (cutoff value of CEA, 9.6 ng/mL) (40% vs. 11%, *p* = 0.0010). Que et al. [51] reported a similar trend, but the cutoff value of the CEA level was 5 ug/L. In NSCLC patients, Cai et al. [25] pointed out that the EGFR mutation frequencies of patients with CEA level < 5 ug/L, 5–20 ug/L, and > 20ug/L were 39.81%, 45.32%, and 65.47%, respectively (*p* = 0.004), indicating a positive association between CEA level and EGFR mutation. Jin et al. [53] found a similar trend in Chinese non-smokers with ADC, and the incidences of exon 19 in those three groups according to the CEA level were 20.9%, 40.7%, and 57.1%, respectively (*p* = 0.007). In contrast, the incidences of exon 21 showed no significant difference in different CEA levels, suggesting that CEA levels may positively correlate with EGFR mutations, especially for exon 19. CEA level can predict EGFR mutation status, and the mutation frequency increases with the elevated CEA level [8,25,26,54]. Therefore, the CEA level can preliminarily predict EGFR mutation status. On the contrary, some studies did not find a correlation between CEA level and EGFR mutation status [6,9,55,56,57,58]. Among the 5780 non-squamous NSCLC patients, no clear trend was found between CEA level and EGFR mutation (*p* = 0.284) [22]. Among common mutations (including 19-Del and L858R) and other mutations, CEA levels were comparable (*p* = 0.162). In a further analysis of different mutation types, 19-Del and L858R mutations also had similar CEA levels [11].

### 4.2. Cytokeratin 19 Fragment

Cytokeratin 19 fragment (CYFRA 21-1) is a fragment of cytokeratin subunit 19, widely used in NSCLC [60]. A large amount of soluble CYFRA 21-1 fragment is released into the blood, increasing the CYFRA 21-1 level in the blood. As a useful tumor marker of lung squamous cell carcinoma, CYFRA 21-1 has high sensitivity and specificity (66.5% and 95%, respectively) [61]. In NSCLC patients, EGFR gene mutation appeared more frequently in patients with the level of CYFRA 21-1 < 2.5 ng/mL (56.77% vs. 46.61%; *p* = 0.005), and EGFR mutation was correlated with CYFRA 21-1 level [9]. Cai et al. [25] suggested that patients with low-level CYFRA 21-1 had a higher rate of EGFR mutation compared to patients with high-level CYFRA 21-1 (cutoff value of CYFRA 21-1, 3.3 μg/L) (*p* < 0.05). Some studies found EGFR mutation frequencies in different CYFRA 21-1 level groups were comparable, suggesting that EGFR mutation did not correlate with CYFRA 21-1 level [6,56,57,59,62].

### 4.3. Squamous Cell Carcinoma Associated Antigen

Squamous cell carcinoma-associated antigen (SCC-Ag) is one of the serine protease inhibitor family of endogenous serine protease inhibitors, mainly existing in the cytoplasm of squamous cell carcinoma of the lung, uterus, esophagus, etc. A high level of SCC-Ag is usually associated with poorly differentiated and advanced metastatic squamous cell carcinoma [63]. Wen et al. [6] indicated that EGFR mutation was more common in NSCLC patients whose SCC-Ag level is under 1.5 ng/mL. That meant the SCC-Ag level was significantly negatively correlated with EGFR gene mutation (*p* < 0.05), and the SCC-Ag level under 1.5 ng/mL was an essential predictor of EGFR gene mutation. Wang et al. [9] also showed a similar trend in their study, and further subgroup analysis of EGFR mutant subtypes (exons 19 and 21) found that SCC-Ag level < 1.5 ng/mL was an important predictor of exon 19 mutation (OR, 0.320; *p* = 0.005). However, Cai et al. [25] reported no statistical difference in the SCC-Ag levels between different mutation points (exon 19 and 20). One hundred and ten patients were detected with the serum EGFR gene mutation status to discuss its correlations with the clinical features, and the results suggested that the level of SCC-Ag in patients having EGFR mutation was lower than the EGFR-wild patients (*p* < 0.05) [64]. Abdurahman et al. [59] held the different view that no significant correlation existed between EGFR mutation and preoperative serum SCC-Ag level in NSCLC patients who received surgical resection (*p* > 0.05).

### 4.4. Serum Ferritin

Serum ferritin is an important protein in human iron metabolism, and abnormal changes may occur in diseases. Serum ferritin levels significantly increased in patients with cancer, especially advanced cancer. In NSCLC patients, its synthesis is highly active with increased levels and can be used as a tumor marker [26]. Existing studies have shown the inconsistent association between serum ferritin levels and EGFR mutations. Wang et al. [9] suggested that in NSCLC patients with low ferritin levels (men < 275 mg/L, women < 204 mg/L), EGFR gene mutations were more common. Wu et al. [26] reported that in NSCLC patients who were in advanced stages, an increased serum ferritin level was more common in patients accompanied with EGFR mutation than EGFR-wild patients (the optimal critical value of serum ferritin for females was 129.0 µg/L and for males 329.0 µg/L, respectively), with a statistically significant difference. They indicated that the level of serum ferritin was able to indicate EGFR mutation status. The higher the serum ferritin level, the higher the mutation frequency of the EGFR gene.

## 5. Liquid Biopsy

### 5.1. Pleural Effusion

Malignant pleural effusion (MPE) refers to pleural effusion caused by the original pleural malignant tumor or metastasis of other malignant tumors involving the pleura. One of the leading causes is lung cancer, accounting for about 1/3 of MPE [65]. About 10%~15% of advanced NSCLC patients are accompanied by MPE when they are initially diagnosed. The proportion of MPE in patients undergoing retreatment is higher, accounting for more than 50% [66]. A growing number of studies suggested that MPE was an effective alternative to detect EGFR mutations. In a study involving 192 NSCLC patients in the advanced stage, 119 (61.98%) matched primary tumor tissues were EGFR mutant, and so were 113 (58.85%) pleural effusion samples. The overall concordance rate of EGFR mutation between two kinds of samples was 86.98%, indicating that pleural effusion may be an effective method to screen EGFR mutation for NSCLC patients in advanced stages [67]. According to a meta-analysis of 1226 East Asian NSCLC patients [68], compared with tumor tissues, the comprehensive sensitivity and specificity of detecting EGFR gene mutation in pleural effusion samples were 0.86 and 0.93, respectively. It suggested that for patients with advanced NSCLC who could not obtain tumor tissue, EGFR gene mutations can be detected by pleural effusion samples instead of tumor tissue. Song et al. [69] performed capture-based targeted sequencing on MPE supernatant and cytological-negative PE (CNPE) supernatant, in which mutation frequencies were 99.2% and 100%, respectively. Therefore, the CNPE supernatant and MPE are comparable in EGFR mutation identification. In addition, EGFR mutation was found in 47.5% CNPE supernatant and 32.5% matched tumor biopsy specimens, indicating that CNPE specimens were better than tumor tissue specimens in detecting EGFR mutations. When Wang et al. [70] conducted a study on MPE, tumor tissue, and plasma samples collected from ADC patients, EGFR mutation rates were 39.3%, 38.0%, and 27.4%, respectively. Compared to tumor tissue specimens, MPE had the sensitivity and specificity of 71.4% and 96.5%, respectively, and the concordance between the two kinds of specimens was 87.1% (Kappa = 0.71).

### 5.2. Circulating Tumor DNA

Circulating tumor DNA (ctDNA) means a fragment of circulating free DNA (cfDNA) released into the blood from the primary tumor or metastatic cells. It carries tumor-specific genetic messages such as tumor-specific point mutations, chromosome rearrangement, copy number variation, and DNA methylation. As a biomarker commonly used for advanced NSCLC, ctDNA is a helpful tool for early detection, stratifying cancer patients, guiding therapy, detecting resistance, and monitoring relapse [71,72]. The consistency of EGFR mutation detection between plasma and tissue samples can reach 75–90% in NSCLC patients [73,74,75,76]. A systematic review and meta-analysis [77] involving 4527 advanced NSCLC patients indicated that compared to tumor tissue, the combined sensitivity and specificity of ctDNA EGFR mutation in plasma samples was 0.70 and 0.98, respectively. That meant ctDNA had high specificity and accuracy in detecting EGFR mutation in advanced NSCLC patients. When patients cannot obtain satisfactory tumor tissue for genetic testing, or the progress of the disease after treatment cannot be monitored by biopsy again, ctDNA genetic testing can help solve these problems. A meta-analysis [78] of advanced NSCLC patients showed that 57% of EGFR mutant patients in tissue samples were ctDNA positive, while 43% were ctDNA negative, suggesting the limitations of EGFR–ctDNA testing. Therefore, when ctDNA gene test results are negative, the risk of misdiagnosis should be considered, and tissue biopsy should be performed to avoid patients meeting the guidelines missing the benefits of EGFR-TKIs [77]. The half-life of ctDNA is short in the blood. Therefore, compared with imaging or clinical symptoms, ctDNA analysis can monitor tumor dynamics in real-time so it can predict treatment response, disease progression, prognosis, and guide clinical decision-making at an early stage [71,75,76]. The prediction of EGFR mutation status by ctDNA was limited by detection technology [79]. Based on the results of tissue detection by cobas, the positive consistency of plasma T790M by cobas, droplet digital PCR (ddPCR), and NGS was 51%, 58%, and 66%, respectively [80]. In addition, ctDNA is also associated with the volume of the primary tumor, aggressive histological characteristics, the size of the largest tumor deposits, disease progression, M stage, and distant metastasis [81,82]. Plasma ctDNA testing has the advantages of non-invasion, instantaneity, and high throughput. With the progress and standardization of genetic testing technology and methods, ctDNA will become an essential part of precision treatment, providing a new way for diagnosing NSCLC and monitoring tumor progression and prognosis.

### 5.3. Circulating Tumor Cells

Circulating tumor cells (CTCs) come from tumor tissue and enter the peripheral blood circulation, which may help detect malignant tumors early [83]. Multiple technologies are available for the detection of CTCs, and a common method is the CellSearch System based on the specific immunological recognition of epithelial cell adhesion molecule (EpCam)-positive cells. In addition, different methods have the different detection frequencies of CTCs. For example, Papadaki et al. [84] reported that the detection frequencies of ≥1 CTCs and ≥5 CTCs by CellSearch were 41.9% and 11.6%, respectively, while the detection frequency of CEACAM5mRNA+ CTCs was 29.3%. Since CTCs analysis is non-invasive, patients can be continuously monitored to provide information such as targeted gene mutations, mutation burden, the incidence of drug resistance, the response of treatment, and prognosis. CTCs analysis has been applied to breast and colorectal cancers and has been proven to have a connection with poor PFS and OS in NSCLC [85]. Jiang et al. [86] pointed out that patients with high CTCs count (CTCs > 17.0 FU/3 mL) had more frequent mutations of EGFR rare genes than patients with low CTCs count (CTCs ≤ 17.0 FU/3 mL) (16.0% vs. 4.9%, *p* = 0.006). Wei et al. [87] found CTCs count in EGFR-mutated patients was higher than that in patients without mutations (*p* < 0.05). Marchetti et al. [88] detected the EGFR mutations by NGS in CTCs specimens from 37 advanced NSCLC patients with EGFR mutations in tumor tissue. EGFR mutations were detected in 31 (84%) of the CTCs preparations, including 25 (81%) exon 19 and 6 (19%) exon 21. Except for higher consistency of EGFR mutation in tumor tissue and CTCs preparations, they also pointed out that the concordances of exon 19 and exon 21 were significantly different (96% and 55%, respectively). A comprehensive analysis involving 7244 patients with lung cancer indicated that to detect EGFR mutations, the CTCs groups had a pooled sensitivity of 75.4% [89]. Sundaresan et al. [90] collected EGFR-mutated patients treated with EGFR-TKIs and compared the T790M genotype by tumor biopsies, CTCs and plasma ctDNA. The study demonstrated that based on tissue biopsy, the concordances of CTCs and ctDNA were 74% and 61%, respectively. While combining the analyses of CTCs and ctDNA, T790M was detected in 37/37 (100%) patients. It indicated that CTCs and ctDNA could be used to predict the drug-resistant genetic mutations and guide the treatment with third generation T790M-selective TKIs.

### 5.4. Non-Coding RNAs

RNA from tumor cells in the blood is called circulating tumor RNA (ctRNAs), including coding RNAs and non-coding RNAs (ncRNAs). NcRNAs are composed of RNAs that have no translation ability, including long non-coding RNAs, micro RNAs, and circular RNAs, which can be used as biomarkers.

#### 5.4.1. Long Non-Coding RNAs

Long non-coding RNAs (lncRNAs) are involved in gene expression, mRNA splicing, and protein subcellular localization through cis and trans mechanisms, and regulate variant malignant processes of NSCLC by epigenetic modification [91,92]. EGFR-TKI resistance, where lncRNAs are involved, is one of the main challenges in the targeted therapy of EGFR-mutant NSCLC. At present, the association between lncRNAs and mutation of the EGFR gene has been confirmed. Lv et al. [93] pointed out that lncRNAs SCARNA7, MALAT1, and NONHSAT017369 were obviously up-regulated in patients who were EGFR mutation-positive compared to patients without EGFR mutation (*p* < 0.05). Among different EGFR mutation types, MALAT1 expression was up-regulated in the 19-Del mutant patients compared to the L858R mutant patients (*p* < 0.05). However, no significant difference existed in the expression of the other two lncRNAs among different EGFR mutation types. The median PFS of patients after the treatment of EGFR-TKIs with high and low MALAT1 levels was 11.6 and 8.2 months, respectively (hazard ratio: 0.431), which meant the high MALAT1 level was related to a better PFS (*p* = 0.020). However, there was no significant correlation between PFS and the NONHSAT017369 expression level (*p* = 0.855) and SCARNA7 expression level (*p* = 0.67). Therefore, lncRNAs may help predict EGFR mutation, response, and prognosis to EGFR-TKIs.

#### 5.4.2. Micro RNAs

Micro RNAs (miRNAs) are a series of non-coding single-stranded RNA composed of about 18 to 24 nucleotides, which are important in gene regulation and involved in tumorigenesis and development processes. Many studies have discussed the influence of MiRNAs on the diagnosis and prognosis of lung cancer [94,95]. A study [96] performed extensive exosomal miRNA analysis on NSCLC patients and noticed that miRNA-1169 could distinguish patients with EGFR gene mutation or not, and the sensitivity and specificity were 80.65% and 91.67%, respectively. In differentiating mutant EGFR from wild-type EGFR patients, miRNA-260 had the sensitivity and specificity of 83.33% and 90.32%, respectively. These results suggested that miRNAs could be predictors of EGFR mutation status. MiRNA-21 is positively correlated with the EGFR gene, which can up-regulate EGFR expression and promote the occurrence and development of NSCLC. Shen et al. [97] reported a study about patients with radical resection of NSCLC. In the EGFR-mutated group, miRNA-21 and miRNA-10b both had significantly increased levels compared to the EGFR-wild group. As for patients with reduced miRNA-21 expression, OS was significantly improved after gefitinib treatment. It indicated that miRNA-21 might be a negative predictor of gefitinib response. In patients with progressive disease, the expression level of MiRNA-10b increased compared to patients with complete remission or disease stability (*p* = 0.001), while miRNA-21 had no prognostic effect on disease progression (*p* = 0.720). However, Szpechcinski et al. [98] found no significant differences of miRNA-10b and miRNA-21 expression level between NSCLC patients with or without EGFR mutations. The plasma miR-504 expression level in EGFR-mutated NSCLC patients was higher than those without EGFR mutations (*p* = 0.0072). It can also be used to predict the EGFR mutation with the sensitivity of 70.37% and the specificity of 82.61%.

## 6. Combined Prediction Models

As mentioned above, several indicators have associations with EGFR gene mutations in NSCLC. Each indicator has its advantages and limitations in predicting EGFR gene mutations, and it is difficult for a single indicator to consider sensitivity and specificity. At present, a number of studies have established a combined detection factor model to indicate EGFR gene mutation status for NSCLC patients by combining multiple predictors [99]. Wu et al. [100] reported a study involving 67 NSCLC patients who were examined by enhanced chest CT before treatment and built the prediction models using clinical features and radiomics features. The AUC under the ROC of clinical characteristics and radionics characteristics was 0.8387 and 0.8815, respectively. Meanwhile, the AUC of the model, which combined clinical and radiomics characteristics, was the highest at 0.9724, with a sensitivity of 85.3% and specificity of 90.9%. The joint model was significantly better than the single clinical or radiomics model, indicating that the joint model was the best to predict EGFR gene mutations. Gu et al. [18] revealed that SUVmax of PET/CT, gender, tumor histology, and CEA were significant indicators for EGFR mutation, and performed a formulation as follows: Y = ex/(1 + ex), X = −8.273 + 1.713 × gender + 1.402 × histology + 0.735 × CEA +0.921 × SUVmax, with the cutoff point 0.3432. When the value was greater than 0.3432, the patient was harboring EGFR mutation, otherwise not. Another study suggested that the AUC for predicting EGFR mutation status based on clinical, general imaging, and radiological characteristics were 0.284, 0.703, and 0.815, respectively, which were all lower than the AUC (0.894) of the prediction model based on the combined three groups of characteristics above [2]. A more intuitive prediction of EGFR mutation was made by establishing a nomogram based on smoking index, pleural contraction, and three radiological characteristics, supported by the C index of 0.894 in the training group and 0.92 in the validation group. In lung adenocarcinoma, Shi et al. [101] developed a nomogram model based on age, gender, smoking history, histological subtypes, Ki67, and radiomics features. This prediction model for EGFR mutation had the diagnostic efficiency of 82.7%. Zhao et al. [102] also demonstrated a model based on 18F-FDG PET/CT radiomics features and clinical factors in identifying EGFR mutations, with a concordance index (C-index) value of 0.841, indicating a good clinical utility.

## 7. Conclusions

EGFR-TKI therapy plays a vital role in NSCLC. Nevertheless, sometimes genetic testing cannot be carried out in clinical application due to difficult access to tissue specimens, limited detection technology, or economic factors. Therefore, it is crucial to predict EGFR gene mutation for patients who cannot undergo genetic testing to determine EGFR mutation status by using indicators and methods such as clinicopathological features, tumor markers, and fluid biopsy. These predictors have the advantages of minor trauma, simple detection method, economy, easy access, and real-time monitoring. On the trend, clinicopathological features (including gender, smoking history, pathological type, histological subtype, and age), immunohistochemistry, degree of differentiation, TMB, STMs (including CEA, CYFRA 21-1, SCC-Ag, and serum ferritin), and fluid biopsy (including ctDNA, CTCs, and ncRNAs) can be used as predictors of EGFR gene mutation. It has guiding significance for precise individualized diagnosis and treatment. However, the application value of a single indicator is limited, so appropriate mathematical models can be established to predict EGFR mutation status by combining multiple indicators in order to improve the clinical value of EGFR mutation prediction.

## Figures and Tables

**Table 1:** Key studies of demographic characteristics and EGFR mutation rates in NSCLC patients.

Reference	Country	Exons Analysed	Patients	EGFR Mutation									
				Age			Gender (%)			Smoking History (%)			
				Median OR Mean Age						Never-Smoker	Smoker		*p*
				Mutant-Type	Wild-Type	*p*	Male	Female	*p*		Former	Current	
Shim [13]	Korea	Exon18–21	ADC	60.4 (42–80)	62.2 (43–77)	0.29	22 (42.3)	32 (58.2)	0.10	37 (56.9)	12 (54.5)	5 (25.0)	0.04
Girard [14]	Non-Asian	Exon18–21	ADC	64 ± 12	64 ± 11	0.370	169 (19.9)	435 (28.2)	<0.001	330 (49.8)	274 (15.8)		<0.001
Girard [14]	Asian	Exon18–21	ADC	60 ± 11	61 ± 12	0.510	97 (50.0)	188 (69.6)	<0.001	226 (68.9)	59 (43.4)		<0.001
Xu [15]	China	Exon18–21	ADC	55.6 ± 9.3	53.7 ± 9.9	0.192	45 (35.7)	57 (59.4)	0.000	74 (61.7)	28 (27.5)		0.000
Dong [16]	China	Exon18–21	ADC	59 (28–76)	57 (23–79)	0.478	35 (36.8)	57 (54.3)	0.016	66 (47.5)	26 (42.6)		0.542
Zhu [17]	China	Exon19,21	ADC	60.5 ± 10.0	55.0 ± 10.5	<0.001	69 (39.4)	151 (37.9)	0.780	184 (39.3)	36 (34.3)		0.375
Gu [18]	China	Exon18–21	NSCLC	58 (52–63)	62 (54–69)	0.064	25 (18.9)	45 (57.7)	<0.001	57 (47.5)	13 (14.4)		<0.001
Grosse [19]	Northeastern Switzerland	Exon18–21	ADC	64.2 ± 13.1	64.1 ± 10.9	0.946	35 (14.9)	55 (23.5)	0.020	69 (60.0)	10 (6.3)	11 (5.7)	<0.001
Wang [9]	China	No data	NSCLC	59 (29–85)	60 (15–85)	0.632	261 (40.9)	284 (63.1)	<0.001	348 (59.7)	133 (33.1)		0.002
Wang [9]	China	No data	ADC	59 (29–85)	59 (25–85)	0.123	252 (45.5)	277 (64.7)	<0.001	337 (62.3)	128 (37.4)		<0.001
Wen [6]	China	No data	NSCLC	66 (59–73)	64 (61–71)	0.512	29 (34.9)	34 (56.7)	0.01	42 (55.3)	21 (31.3)		0.004
Lemine [20]	Morocco	Exon18–21	NSCLC	61	62	0.041	35 (14.5)	38 (41.4)	<0.0001	47 (354.8)	23 (13.0)		<0.0001
Nakra [21]	India	Exon18–21	NSCLC	55 ± 12.2	57 ± 12.4	>0.05	148 (23.0)	121 (45.7)	0.001	103 (57.5)	52 (28.4)		<0.001
Suda [22]	Japan	Exon19 Del, L858R, or others	nonsquamous NSCLC	67.7 (67.3–68.1)	67.6 (67.2–67.9)	0.612	826 (27.1)	1584 (58.2)	<0.001	1552 (59.8)	529 (30.2)	262 (21.2)	<0.001

Abbreviations: EGFR, epidermal growth factor receptor; NSCLC, non-small cell lung cancer; ADC, lung adenocarcinoma.

**Table 2:** Key studies of serum tumor markers and EGFR mutation rates of NSCLC patients in East Asia.

Reference	Exons Analysed	Patients	EGFR Mutation											
			CEA (%)			CYFRA21-1 (%)			NSE (%)			SCC-Ag (%)		
			Negative	Positive	*p*	Negative	Positive	*p*	Negative	Positive	*p*	Negative	Positive	*p*
Jin [53]	Exon18–21	ADC	24 (55.8)	43 (78.2)	0.018	52 (67.5)	15 (71.4)	0.734	59 (69.4)	8 (61.5)	0.570			
Yang [8]	Exon19,21	NSCLC	27 (18.8)	81 (47.4)	<0.05									
Abdurahman [59]	Exon18–21	NSCLC	5 (27.8)	20 (60.6)	0.025	10 (55.6)	15 (45.5)	0.346				23 (47.9)	2 (66.7)	0.610
Lu [58]	Exon18–21	ADC	84 (60.4)	48 (60.0)	1.000									
Cai [25]	No data	NSCLC		35 (57.4)	0.014	70 (43.5)	32 (36.0)	0.01	71 (43.0)	31 (34.8)	0.36	98 (44.7)	4 (18.2)	<0.001
Zhu [17]	Exon19,21	ADC	133 (35.2)	87 (44.6)										
Wu [26]	No data	NSCLC	49 (41.5)	109 (59.6)	0.002	76 (48.7)	82 (56.6)	0.204	93 (51.7)	65 (53.7)	0.408			
Wen [6]	No data	NSCLC	15 (34.1)	48 (50.0)	0.079	17 (38.6)	46 (48.4)	0.281	34 (44.2)	29 (46.8)	0.873	48 (51.1)	6 (26.1)	0.031
Wang [9]	No data	NSCLC	211 (47.4)	329 (52.6)	0.092	172 (56.8)	234 (46.6)	0.005	187 (54.2)	195 (69.6)	0.214	388 (52.2)	50 (33.6)	<0.001
Wang [9]	No data	ADC	204 (53.7)	321 (54.7)	0.760	170 (60.7)	222 (50.3)	0.006	182 (58.0)	184 (53.0)	0.202	378 (55.4)	46 (40.7)	0.004

Abbreviations: EGFR, epidermal growth factor receptor; NSCLC, non-small cell lung cancer; CEA, carcinoembryonic antigen; CYFRA21-1, cytokeratin 19 fragment; NSE, neuron-specific enolase; SCC-Ag, squamous cell carcinoma-associated antigen.
